# Rethink space: (Re)designing a workspace using human-centered design to support flexibility, collaboration, and engagement among clinical and translational research support services

**DOI:** 10.1017/cts.2017.5

**Published:** 2017-08-10

**Authors:** Aalap Doshi, Christina Clay

**Affiliations:** 1 Michigan Institute for Clinical and Health Research, University of Michigan, Ann Arbor, MI, USA; 2 The Brehm Center, University of Michigan, Ann Arbor, MI, USA

**Keywords:** Human-centered design, collaboration, workspace, research support, engagement

## Abstract

**Introduction:**

Space matters. We read space like we read people’s faces. Space is an instrument of collaboration and innovation. At the University of Michigan’s Institute for Clinical and Health Research (MICHR), a team was created to creatively and economically enhance our operating space into a flexible workspace that supports privacy, innovation, creativity, and most important, a culture of collaboration.

**Methods:**

The team used a human-centered design process to creatively engage the staff at large into analyzing our existing space, identifying latent needs, proposing solutions, generating feedback, and economically building the rethought process.

**Results:**

The redesigned workspace embraces the differences among MICHR’s teams while encouraging collaboration and teamwork and keeping costs at a minimum. It has resulted in a flexible space that includes co-located teams, spaces dedicated to different work goals, an open area for collaboration, quiet zones for focused work, and better wayfinding.

**Conclusions:**

Through our Rethink Space project, we hope to have demonstrated that, by initiating the project internally and by engaging the users of the space themselves in an empathetic, visual, and human-centered way, a space redesign can be undertaken economically while also leading to improved levels of employee and team satisfaction.

## Introduction

Space matters. We read space like we read people’s faces. Space is the foundation for the expression of our cultural values. Space is an instrument of collaboration and innovation [1]. Situating relationships in space is instrumental to formulating better models of collaboration and information sharing in organizations. In the paper titled “Shared paths to the lab: a sociospatial network analysis of collaboration,” Kabo *et al*. [2] have suggested that a design in which male and female bathrooms are situated on the opposite ends of a long hallway will cause an increase in the rate of same-sex collaborations while decreasing the incidence of mixed-sex collaborations.

At the University of Michigan’s Institute for Clinical and Health Research (MICHR), we realized that our workspace was having an impact on how we were functioning. MICHR is 1 of the 62 academic research institutions that have received a Clinical & Translational Science Award, which aims to accelerate discoveries toward better health. MICHR has over 100 employees functionally organized into more than 12 programs. In the 2013 MICHR employee engagement survey results, MICHR staff commented on challenges with the environment in which they performed their work. Some of these challenges included being physically separated from other team members, limitations on meeting space including size and availability and no lunch or break space nearby. The building was laid out with offices around the perimeter and all cubes in the center. With conference rooms open to everyone at the University finding meeting space was difficult. In response to these challenges, MICHR’s Managing Director created a team of volunteers to study the issue of space and provide recommendations on how our physical space could help us improve communication, collaboration, productivity, and engagement.

This article outlines the process this team used to creatively engage the staff in understanding and analyzing our existing (now old) space, identifying latent user needs, proposing solutions, and building the new space. It hopes to demonstrate that by engaging the users themselves (in our case, the MICHR staff), a space redesign can be undertaken economically while also leading to improved levels of employee and team satisfaction. Finally, we propose that the empathetic, visual, human-centered process we used to redesign our space can be applied to other complex problems that have fuzzy goals.

Most complex problems today have extremely fuzzy goals. Authors Gray *et al*. in their book on “Gamestorming” observe that success in industrial work involves consistent, repeatable results whereas success in knowledge work involves breakthrough ideas [3]. For something to be innovative, it needs to be new, surprising, and radically useful [4]. If we want to create new and innovative solutions, there is no way to define the goal in advance, because there are too many variables. We need to imagine a future world, one that we cannot fully conceive yet. Our goals need to be fuzzy. Cambridge Professor Alan Blackwell and colleagues identified a fuzzy goal (they called it a polar-star vision) as one that motivates the general direction of work without blinding the team to opportunities along the journey [5].

Rethinking our space was a problem with a fuzzy goal. While we as an organization had a vague, nebulous idea of what we wanted our space to embody and stand for, there were too many unknowns to have any specifics. Our project involved creating a platform to support exploration, experimentation, and progress toward our fuzzy goal.

## Methods

### The Redesign Process

The “Rethink Space Team” used a human-centered design process to engage the MICHR staff to redesign our space. The Human Centered Design Toolkit defines human-centered design as a process and a set of techniques used to create new solutions for the world [6]. It goes on to say that the reason it is called “human centered” is because it starts with the people you are designing for. The process helps you hear the needs of the constituents in new ways, create innovative solutions to meet those needs, and deliver solutions with financial sustainability in mind. The toolkit describes a successful solution at the end of a human-centered design process as hitting the overlap between desirability, feasibility, and viability. While a generally accepted definition of design thinking has yet to emerge, Thomas Lockwood, former President of the Design Management Institute, a leading association of design practitioners working in business, has offered perhaps the most detailed definition of design thinking: “A human-centered innovation process that emphasizes observation, collaboration, fast learning, visualization of ideas, rapid concept prototyping, and concurrent business analysis” [7].

This process involved 4 overarching phases—Hear, Understand, Create, and Deliver. These phases are explained below.

### Hear: Assessment of Staff and Organizational Needs

This phase was all about generating empathy. We wanted to understand people and their lives at MICHR. We wanted to see them at work, hear what they hear, feel what they feel, and know what they think. Below is how we went about it.

#### Idea Board

One of the first things we did as a team was to create a physical board where others could raise their grievances, make suggestions, and list inspirations. It helped us open up to our staff and set the expectations for co-creation.

#### In-Context Immersion

To gain empathy and understand the people we were designing for on an intellectual, emotional, and experiential level, we immersed ourselves in their cubes, offices, meeting places, hallways, and storage places. We observed what they did and how they did it, and asked them questions about why they did what they did. We took photographs of their workspace and noted our conversations. We uncovered great insights and many latent opportunities from understanding people in their context.

#### Co-create Workshops

We organized various workshop-style events to involve everyone working at MICHR in designing their own workspaces. These events involved fun activities centered around understanding and co-creating space and culture. Not only did they help us peel the layers off the existing culture, they also brought everyone together and spread awareness about the importance of space in our work lives.

Some of the artifacts generated out of these workshops include the following.

#### Network Map

**Fig. 1:**
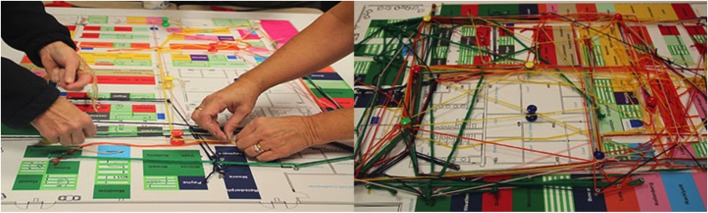
A network map being built. The network map helped us understand how our space was being traversed.

To understand how we as an organization traverse our space, we invited the MICHR staff to co-create a network map (color-coded by working group) that mapped the most-traveled routes within the organization. As you can see in Fig. 1, the exercise helped us become aware of our regular paths and opened our eyes to how physically trapped we were within our own groups.

#### Low-Tech Social Network

To map out the connections between people, we invited the staff to create a low-tech social network. Members added themselves as nodes in the network and then drew connections between themselves and their peers with whom they interacted.

The low-tech social network made us realize that the administrative assistants are the most connected nodes in the system and are central to space and information flow within the organization. The network also helped us identify connections between people we would have normally not guessed.

#### Cover Story

To understand the aspirations of the MICHR staff in relation to their workspace, we asked them to imagine that the space was already built and was so successful that *Time* magazine was doing a cover story on it. We then asked them to imagine for us what the header, sections, images, and callouts of the cover story would be.

This imaginary scenario freed the staff to think without the constraints of budgets and realistic possibilities and brought out the inner visions they had for their space.

#### Empathy Map

In order to quickly develop a user profile and create a shared understanding among the staff of their colleagues existing state, we worked with them to create an “empathy map.” This involved brainstorming what John Doe, a representative MICHR staff member, thinks, feels, does, sees, and hears when he enters the MICHR space.

#### Ideation Sessions

To get a deeper understanding of our staff’s latent and as yet unexpressed needs, in each of the workshops we broke into smaller working groups to think through and prototype space-related solutions. Some of these workshops focused on broader problems whereas others focused on more specific ones. As a way of achieving some convergence on the ideas that had been brainstormed, the finished solutions were then posted and voted on by the larger group.

The solutions that the staff came up with gave us a good understanding of what they were expecting to achieve at the end of the project and gave us insights into what some of their needs were. Voting on ideas not only helped spread the ideas but also acted as a consensus-building exercise. We then used these as inputs into our final design.

### Understand: Analyze Findings

This phase involved surfacing themes from all the user research that had been conducted as described above. It also involved spotting opportunities and converting them into stories that could be shared back with our staff.

In order to externalize the knowledge we had gained from the “Hear” phase and use it in our discussions, we built an affinity diagram and posted it on our team room walls. Holtzblatt *et al*. describe an affinity diagram as follows: “The affinity diagram organizes the individual interpretation session, or affinity, notes into a wall-sized hierarchical diagram grouping the data into key issues under labels that reveal the customers need. The affinity shows in one place the common issues, themes and scope of the customer problems and needs” [8].

We also displayed within our team room the various artifacts that were generated from the “Hear” phase. They constantly reminded us of all that we had learned and made sure that we were always thinking about our research. We also opened the team room to all MICHR staff, promoting transparency and co-ownership in the process.

The affinity diagramming exercise and insights generated from the other artifacts led us to see the emergence of the following high-level themes for staff and organizational needs the following.

#### Spatial Colocation Within Teams

**Fig. 2:**
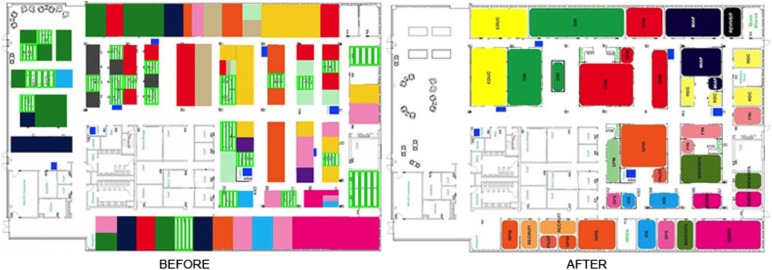
Map of existing and proposed University of Michigan’s Institute for Clinical and Health Research program locations.

As you can see in Fig. 2, where colors denote teams in a network map, MICHR’s staff was spread out in an unsystematic manner. People from a program/team were not always spatially colocated. This was causing inefficiencies and was leading to a lack of communication within the programs. It was also adversely impacting team morale.

#### Flexible Workspaces

As described in the introduction to this article, MICHR has a variety of units that do very different work but need to collaborate often. To support the roles that we play every day at MICHR, we needed (a) flexible work spaces that support small and large areas to come together for groups of various sizes, (b) extended horizontal work surfaces for jobs such as creating packets for a grant or an educational workshop, (c) vertical work surfaces for project management, (d) program-specific spaces for programs such as “Clinical Research Informatics (CRI),” and (e) quiet spaces where we could focus.

#### Wayfinding

MICHR occupies 1 building within a suite of buildings. We were organized as rows of cubes and there were few landmarks within our space for guidance. People external to MICHR, such as investigators and research team members, were often lost in our space and stopped by at random staff cubes asking for directions to major meeting rooms.

#### Noise Level

Our staff thought the entire MICHR space was very quiet. They equated it to a library and assumed that as in a library, the normal mode of operation in our entire space was to be quiet and work alone. They did not feel comfortable going up to someone and asking a question, as it would disturb that person and others in the area. This was leading the staff members to be very conscious of communicating with each other.

#### Space Ownership

We realized there was a lot of uncertainty within the staff over who owned which piece of space and what could or could not be done in the space. This led most staff members to feel reluctant to experiment with and use the space we had to suit their needs.

### Create: Propose Solutions and Get Feedback

In this phase we used the insights gathered from the “Hear” and “Understand” phases to propose an alternative workspace design for MICHR. We then iteratively shared this proposal with our staff and made improvements to it based on their feedback. Finally, we conducted a poll asking all our staff whether they were willing to move ahead with the space redesign and promised them we would only implement the new design if a majority were in favor.

#### Guiding Principles

Culture theorists going as far back as Émile Durkheim have argued that shared beliefs, norms, and values shape people’s thoughts and behaviors. With that in mind, one of the first things the Rethink Space team did in this phase was to create a series of guiding principles based on our design research that could guide us in redesigning our space. Our guiding principles are listed below:

∙ Enhance, not replace;
∙ Make space for change;
∙ Leave room to evolve;
∙ Design for social collisions;
∙ People, not technology;
∙ Bold is better than bland;
∙ Small changes can have a profound impact.

#### Proposed Space Redesign

The maps in Fig. 2 show MICHR’s old space and the redesigned space we proposed. The colors denote the location of the different programs within MICHR.

Below are some highlights of the redesigned space:

#### Zones

**Fig. 3:**
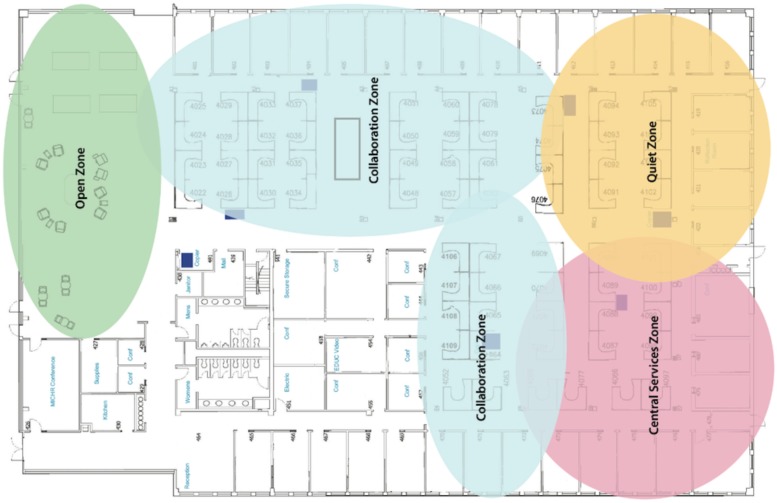
University of Michigan’s Institute for Clinical and Health Research space divided into zones.

Zoning is a device of land-use planning used by urban planners around the world to designate permitted uses of land based on mapped zones. We loosely adopted the concepts of zoning from urban planning to create zones at MICHR that would guide us in planning our space. As shown in Fig. 3, we created 4 different zones to match staff and organizational needs. These zones were based on various parameters including noise levels; proximity to the main hallway; proximity to the courtyard, kitchen, and restrooms; and the type of work performed.

The 4 zones are detailed below.

(1) Open zone: the open zone was established primarily to support the need for formal and informal gathering spaces for groups of all sizes. The open zone is meant for temporary interactions and is not meant to be a permanent home for any of our staff.
(2) Collaboration zone: the collaboration zone was set up to house MICHR programs like “CRI” and “Clinical Research Management Services” that needed close collaborative teamwork as their default mode of operation.
(3) Quiet zone: the quiet zone was established to house MICHR services like “Grant Writing Support” and “Regulatory Support” that needed quiet, focused environments as their default mode of operation.
(4) Central services zone: the central services zone was established to house MICHR’s internal services such as “Human Resources” and “Finance.”

#### Colocation

As discussed in the “Understand” phase above, MICHR’s staff was spread out in an unsystematic manner. As can be seen from the maps in Fig. 2 where each color represents a different MICHR program, the proposal aimed at getting teams spatially together to boost productivity, communication, and morale.

#### Cube Configuration

Through our Hear phase, we realized that a pod-like cube configuration worked best for programs at MICHR. It allowed people to turn around and communicate with their team members, it made impromptu conversations possible, and helped to uncover and solve problems faster. In our old space, only about half of our cubes were set up as pods.

As part of our redesign, a pod of cubes became our basic building block. We expanded on this unit to offer as many pods as possible to aid with communication and collaborations within teams.

#### Dedicated Team Spaces

Our design research helped us see that there are teams across MICHR that need dedicated spaces to come together to perform specific tasks such as assembling packets, brainstorming, and project management. The proposed design included assigning offices to teams that needed them to be used as team rooms.

The CRI program’s development team at MICHR creates digital products. We learned that their efficiency and quality of work was considerably boosted when they had an open space that matches the way in which they function. The proposed design included creating an area within the CRI space dedicated to digital development.

#### Open Area and Quiet Space

The proposed open area was a flexible space in the open zone where staff could have lunch with their friends, grab their peers and collaborate, stand and work, organize and participate in events, or simply enjoy beautiful views of the courtyard. The open area would be created by consolidating the open, unused cubes, and making the area by the courtyard available to all. Technology and prototyping materials would be introduced in the space to provide for random collaborations and a bias toward action.

To balance the open active spaces around MICHR, the proposed design also contained a shared quiet space in the quiet zone. The quiet space would be available for anyone to walk into when they need quiet focused time to work or reflect.

#### Wayfinding

The proposed design allowed us to come up with multiple wayfinding mechanisms to help MICHR employees and visitors find people and resources at MICHR. These included branding signs identifying programs in their space, directional signs directing people to various programs and services, and utility related signs like “Kitchen” and “Supplies.”

#### Feedback

To get feedback on our proposal, we first presented the proposal to each program individually. We did so in our team room where we walked them through our process and heard from them their thoughts and feelings about the proposed redesign. We took their feedback and reiterated our design before presenting it to the next program. We did so until all the programs had been presented to and heard from.

To account for the people who did not want to provide us feedback in a group, we then posted the proposal on a wall in the lobby. We encouraged everyone to take a look at it and leave us feedback on the pink sticky notes provided. We responded to each thought using a yellow sticky note. At the end of our predefined time period, we took the feedback we received and incorporated it into our final plan.

#### Poll

At the end of our feedback period, we conducted a poll to determine the staff’s backing of the proposal. The poll contained the question “Do you support moving forward with the space redesign of NCRC [North Campus Research Complex] 400?” and a space for the staff to provide comments if they so wished. The poll results showed a majority backed the proposal.

### Deliver: Implementation of Findings

This phase involved planning and building, economically and within the given timeframe, the new space that we had proposed in the “Create” phase.

#### Moves and Construction

To meet our new proposal, we had to move a total of 70 staff members. We planned the moves so as to not disrupt any MICHR services. MICHR was not closed for the moves and the moves took place on weekdays during regular business hours. The moves were distributed sequentially across 30 days, with every staff member having an average of 2 days to move. The plan was based on already available open spaces, availability of construction crews, construction noise, and program-specific considerations like large meetings that would cause staff to be out of the office. To lighten the stress of moving, on the first day in their new space, the staff member would find welcoming them a thank you note attached to a bag of candies.

#### Furniture

To keep the costs to a minimum, most of the needed furniture was reclaimed from unused buildings in the immediate research complex or from the University of Michigan’s Property Disposition Center.

#### Signage

Signs were placed at strategically important locations to help with wayfinding, branding, and team morale.

#### Budget

The majority of our $35 000 budget was spent on construction, whiteboards, monitors, and furniture for the open space. Notably, we were able to repurpose unwanted furniture from other units housed at our complex.

## Results

**Fig. 4:**
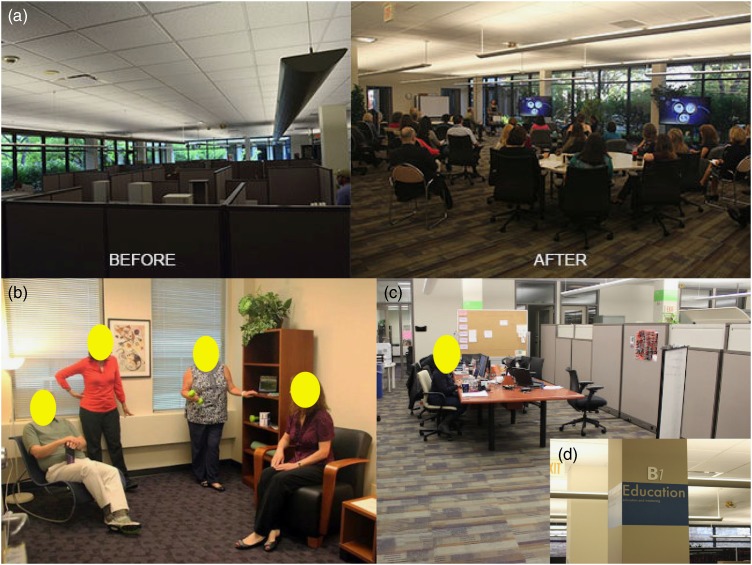
A snapshot of resultant spaces. (*a*) Area by the courtyard before and after the redesign process. (*b*) Quiet space for wellness and reflection. (*c*) I.T. development and innovation space. (*d*) A signage example.

Our staff-led redesign processes led us to the new spaces shown in Fig. 4.

### Continuing Assessment of Space Use, Collaboration and Impact on Organizational Culture

Organizational culture has been variously defined. Little agreement exists over a precise definition of organizational culture, how it should be observed or measured, or how different methodologies can be used to inform routine administration or organizational change [9]. It follows that there is precious little research exploring the impact of space on organizational culture.

That being said, in the short time following the opening of the new space, the Rethink Space team has received positive anecdotal feedback from MICHR staff and guests suggesting that the new space is “more functional,” “matches their style of work,” “is more collaborative,” “is less intrusive,” “has more natural light,” and “is friendlier.” The project was nominated for a Medical School Administration team award by the staff. The team also won an Ergo Hero award from the University of Michigan for our creative efforts in building a multiple purpose open space, thus improving communication and collaboration among staff. In terms of usage, we have observed that the open space is almost always occupied, either by a single large group or by multiple smaller teams. Attendance seems to have increased at MICHR events like the monthly all-staff meetings, lunchtime enrichment presentations, and celebrations, which are now all held in the open space. The quiet area is also occupied often and the dedicated team rooms are in use.

### Survey Results

Six months after the reconfiguration of our space, we surveyed staff to get their direct feedback on the changes. Through observation and anecdotal collection, we felt the changes were overall positive and successful. The survey gave us more concrete information on how things were really going.

The 10-question survey was taken anonymously with an option to leave contact information at the end. The survey included both ratings and open-ended questions. We had a 60% response rate. Overall satisfaction with the new collaboration space was good, with an average rating of 4.26 out of 5. Responders commented, “It’s a flexible space that no one ‘owns.’” “This creates a more inviting and welcoming atmosphere where people can come together to visit, collaborate and create synergy” and “It has really helped with team dynamics.” Satisfaction with individual workspaces was an average of 3.83 out of 5 and satisfaction with team rooms was 3.61 out of 5, noting that not everyone has a team room.

We also asked about satisfaction with the process itself. Responses average 4.13 out of 5. Numerous comments were left, providing positive feedback and suggestions for improvements.

## Discussion

Flexibility, collaboration, and staff engagement are important characteristics that define successful organizations of the 21st century. Space is the platform on which face-to-face social interactions and the networks that result from them are enacted [1]. Space has an impact, however subtle or hard to measure, on organizational culture. Through our Rethink Space project, we hope to have demonstrated that by initiating the project internally and by engaging the users of the space themselves in an empathetic, visual, and human-centered way, a space redesign can be undertaken economically while also leading to improved levels of employee and team satisfaction. This project from concept to implementation took ~1 year. Going forward we intend to continue to review our space design to facilitate even bigger impacts on flexibility, collaboration, efficiency, and engagement in order to further MICHR’s mission to enhance and enable clinical and translational research through the clinical and translational research support services we provide.

Finally, we as the Rethink Space team have found immense joy and satisfaction working on this project and have learned a lot about how to approach problems that have nebulous goals. *We believe that we have the beginnings of a framework for exploration, experimentation, and trial and error that can help solve complex problems that have fuzzy goals*. Whether you are redesigning your workspace, rethinking a service that you offer, or reimagining a more just world, we believe the empathetic, visual, human-centered process we have experienced deserves consideration. More detailed information on our process is outlined on the Rethink Space Web site at http://www-personal.umich.edu/~aalapd/new-website/index.html
